# Structural Insights into Streptococcal Competence Regulation by the Cell-to-Cell Communication System ComRS

**DOI:** 10.1371/journal.ppat.1005980

**Published:** 2016-12-01

**Authors:** Antoine Talagas, Laetitia Fontaine, Laura Ledesma-Garca, Johann Mignolet, Inès Li de la Sierra-Gallay, Noureddine Lazar, Magali Aumont-Nicaise, Michael J. Federle, Gerd Prehna, Pascal Hols, Sylvie Nessler

**Affiliations:** 1 Institute for Integrative Biology of the Cell (I2BC), CEA, CNRS, Univ. Paris-Sud, Université Paris-Saclay, Gif-sur-Yvette cedex, France; 2 Institute of Life Sciences (ISV), Biochemistry, Biophysics and Genetics of Microorganisms (BBGM), Université catholique de Louvain, Croix du Sud, 4-5 (L7.07.06), Louvain-La-Neuve, Belgium; 3 Dept. of Medicinal Chemistry and Pharmacognosy, Center for Biomolecular Sciences, University of Illinois at Chicago, Chicago, Illinois, United States of America; 4 Department of Microbiology and Immunology, University of Illinois at Chicago, Chicago, Illinois, United States of America; 5 Center for Structural Biology, Research Resources Center, University of Illinois at Chicago, Chicago, Illinois, United States of America; National Jewish Health, UNITED STATES

## Abstract

In Gram-positive bacteria, cell-to-cell communication mainly relies on extracellular signaling peptides, which elicit a response either indirectly, by triggering a two-component phosphorelay, or directly, by binding to cytoplasmic effectors. The latter comprise the RNPP family (Rgg and original regulators Rap, NprR, PrgX and PlcR), whose members regulate important bacterial processes such as sporulation, conjugation, and virulence. RNPP proteins are increasingly considered as interesting targets for the development of new antibacterial agents. These proteins are characterized by a TPR-type peptide-binding domain, and except for Rap proteins, also contain an N-terminal HTH-type DNA-binding domain and display a transcriptional activity. Here, we elucidate the structure-function relationship of the transcription factor ComR, a new member of the RNPP family, which positively controls competence for natural DNA transformation in streptococci. ComR is directly activated by the binding of its associated pheromone XIP, the mature form of the *comX*/*sigX*-inducing-peptide ComS. The crystal structure analysis of ComR from *Streptococcus thermophilus* combined with a mutational analysis and *in vivo* assays allows us to propose an original molecular mechanism of the ComR regulation mode. XIP-binding induces release of the sequestered HTH domain and ComR dimerization to allow DNA binding. Importantly, we bring evidence that this activation mechanism is conserved and specific to ComR orthologues, demonstrating that ComR is not an Rgg protein as initially proposed, but instead constitutes a new member of the RNPP family. In addition, identification of XIP and ComR residues important for competence activation constitutes a crucial step towards the design of antagonistic strategies to control gene exchanges among streptococci.

## Author Summary

Bacterial cell-cell communication systems are based on the secretion of signal molecules. These quorum-sensing systems allow bacteria to coordinate genes expression according to the density of their local population. In Gram-positive bacteria, intracellular quorum sensors regulated by re-internalized signal peptides control the expression of genes involved in essential bacterial processes such as horizontal gene transfer, biofilm formation, sporulation or virulence. In most streptococci, including pathogenic species, the ComR regulator and its cognate signal peptide ComS activate competence for natural DNA transformation, a major mechanism for horizontal gene transfer and antibiotic resistance acquisition. To elucidate the molecular mechanism of ComR activation, we performed the structure-function analysis of the ComRS system from *S*. *thermophilus*. We solved the crystal structures of the apo form of ComR and of the complex with XIP, the mature form of ComS, and DNA. We showed that peptide binding shifts the protein from an inactive monomeric state, characterized by the sequestration of the DNA-binding domain, to an active dimer. This idiosyncratic mechanism was confirmed by *in vitro* interaction measurements and *in vivo* transcription assays using ComR mutants and XIP variants. These results may be of special interest for the future design of new antimicrobials targeting antibiotic-resistant pathogens.

## Introduction

Within Gram-positive bacteria, cell-to-cell communication processes, also called quorum-sensing (QS), are regulated *via* oligopeptidic pheromones[1, 2] whose production and secretion are initiated in response to specific environmental stimuli or stresses [1, 2]. At a threshold extracellular concentration of pheromones, QS systems are activated, which ultimately coordinate the expression of target genes in the (sub)population [3]. Secreted pheromone peptides are matured by species-specific proteases and either detected in the medium by the extracellular receptor domain of histidine kinases of two-component systems or reinternalized through oligopeptide permeases for direct interaction with intracellular receptors [4]. The first identified examples of cytoplasmic QS regulators were grouped in a superfamily called RNPP (for the original members Rap/NprR/PlcR/PrgX) [5]. They regulate important physiological processes such as sporulation [6, 7] and virulence [8] in bacilli or conjugation in enterococci [9]. They are characterized by a tetratricopeptide-repeat (TPR)-type peptide-binding domain containing 5 to 9 TPR motifs. Each motif is a degenerate 34-amino acid repeated sequence forming two antiparallel α-helices. The TPR consensus sequence defined by a pattern of small and large amino acids is poorly conserved and TPR motifs are difficult to predict. Their repetition forms a right-handed super-helical domain involved in pheromone binding and dimerization [10]. Except for the Rap phosphatases, RNPP proteins are transcription factors displaying an N-terminal HTH domain, which binds to specific DNA motifs [11, 12]. Structure-function studies have been undertaken for all members of the family: PrgX [13, 14], PlcR [5, 15], Rap [16–19], NprR [20, 21]. In all cases, pheromone binding to the TPR domain induces conformational changes that regulate their activity. However, the induced molecular rearrangements and the detailed molecular mechanisms of protein activation/de-repression greatly differ among RNPP regulators.

Recently, pheromone-interacting transcriptional regulators, named Rgg, have been identified and included in the RNPP family. Two phylogenetic Rgg sub-clusters have been defined: the small hydrophobic peptides (SHP)-associated Rgg and ComR regulators [22, 23]. They are widespread in Firmicutes and in particular in streptococci, where they regulate many important biological processes such as biofilm development [24], antimicrobial resistance [25], or virulence [26]. The structure of Rgg2 from *Streptococcus dysgalactiae* has been solved, alone and in complex with a cyclic peptide antagonist [27]. Recently, a low-resolution structure of the TPR domain of RopB from *Streptococcus pyogenes*, a subgroup of Rgg regulators, has also been published [28].

ComR regulators regulate competence in streptococci [29]. They are activated by ComS signaling peptides, which after maturation and reimport, interact with the ComR regulator. The resulting complex specifically recognizes pseudo-palindromic 20-bp DNA sequences called the ComR-box [30]. The ComR regulon includes the *comS* and *comX* genes. The latter encodes the alternative sigma factor σ^X^, which positively regulates genes required for DNA transformation [30–33]. The mature form of ComS, which corresponds to the C-terminal part of the peptide, is thus called XIP for *comX*/*sigX*-Inducing Peptide [34]. Based on the sequence of XIP peptides, the ComRS systems have been classified into three distinct types [35]. Type I XIP peptides are found in streptococci from the salivarius group and are characterized by the presence of a (V/L)P(F/Y)F motif. They contain no charged residues (e.g. *Streptococcus thermophilus*) whereas XIP peptides from type II, that are found in groups bovis, pyogenes and mutans, contain a WW motif and in some cases, a basic or acidic residue, or both (e.g. *Streptococcus mutans* and *Streptococcus pyogenes*). Because most XIP peptides from *Streptococcus suis* strains do not display two contiguous aromatic residues and are rather characterized by a WG(T/K)W motif, they were classified as type III [35].

Competence for DNA transformation contributes to genome plasticity through stable acquisition/loss of genetic material and plays a dominant role in driving the evolution and survival of microbial populations [36–38]. In particular, this mechanism allows bacteria to acquire and share genes encoding antibiotic resistance [39]. In addition, competence activation in streptococci also results in the co-expression of genes involved in virulence-associated functions and predation mechanisms, which are essential for their fitness during infection [40, 41]. Inhibiting competence development thus constitutes an interesting target in drug discovery. Studies have already been undertaken [42], which target the well characterized two-component signaling system ComDE. The latter also regulates competence in *Streptococcus pneumoniae* via σ^X^ in response to the extracellular Competence-Stimulating-Peptide (called CSP) [43]. However, targeting the ComRS system has not been explored yet.

In this manuscript, we present the structure-function analysis of the ComRS system from *S*. *thermophilus* and provide the structural basis required for rational design of inhibitors. We reveal an idiosyncratic regulatory mechanism based on the sequestration of the DNA-binding domain. Based on protein sequence conservation, we also suggest that this original mechanism is conserved among ComR orthologues. Finally, we discuss how these results might help understand the specificity of the system and control of competence development in pathogenic streptococci.

## Results

### The ComR/XIP/DNA ternary complex is a flexible dimer

**Fig 1:**
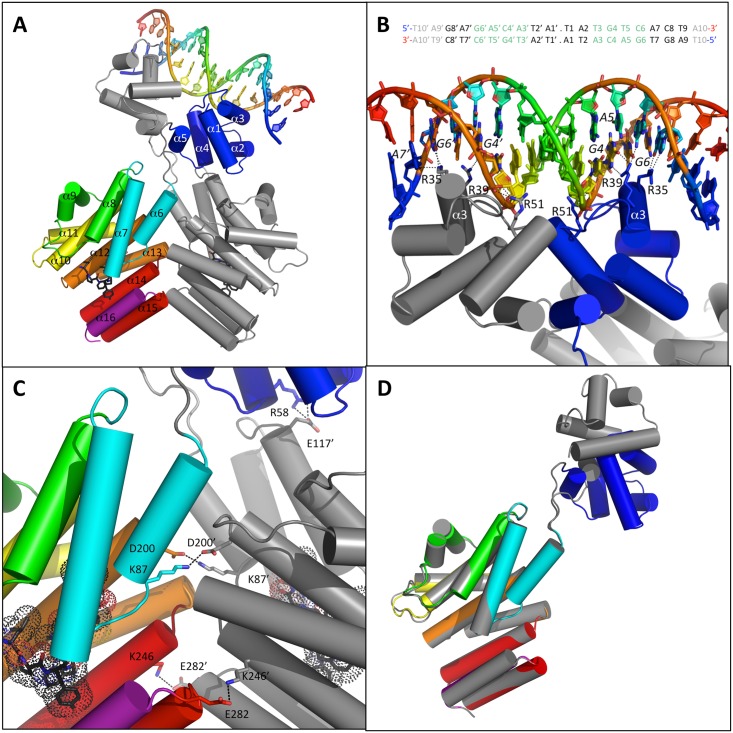
Crystal structure of the ComR/XIP/DNA ternary complex. **A—Overall structure of the complex.** The ComR dimer is shown as cartoon with chain A in gray and chain B colored by spectrum from TPR-1 in cyan to TPR-5 in red with the additional CAP helix α16 in purple and the HTH-domain in blue. The α-helices are labelled. The 20-bp DNA fragment from the *comX* promoter is shown as cartoon colored by spectrum, from 5’ in blue to 3’ in red. The bound peptides are shown as black sticks. **B–Close view of the HTH-DNA interaction.** Same representation as panel A. The protein residues R35, R39 and R51 are highlighted in sticks colored by atom type and labeled. The DNA bases of the conserved GACA motif are colored by atom type. The nucleotides directly interacting with the protein are labeled. The sequence of the crystallized 20-bp DNA fragment is shown at the top of the panel with the conserved GACA motif highlighted in green. The flexible extremities not visible in the electron density map are indicated in gray. The base pairs are numbered from 1 to 10 in each half-site of this pseudopalindromic sequence. **C—Close view of the TPR dimerization interface**. Chain A in gray and chain B colored by spectrum. Residues K87, D200, K246 and E282 involved in stabilizing salt bridges are highlighted as well as residue R58 from chain B and E117 from chain A. **D—Superimposition of the two TPR domains.** Chain A in gray and chain B colored by spectrum.

The structure of the complex between full-length ComR from *S*. *thermophilus* LMD-9, the octapeptide XIP (ComS_17-24_ LPYFAGCL) and the pseudopalindromic 20 bp ComR-box from the *comX* promoter (5’-**T10-A9-G8-**T7-**G6-A5-C4-A3-T2-A1.T1’-A2’-T3’-G4’-T5’-C6’-**T7’-**C8’-T9’-A10’**-3’) has been determined at a resolution of 2.5 Å. The crystals diffracted in space group C2 with a dimer of the ComRS complex bound to one molecule of dsDNA per asymmetric unit (Fig 1A). Only 17 base pairs were visible in the electronic density map. According to a W3DNA analysis [44], the dsDNA fragment displays an almost typical B conformation (3.28° deviation compared to standard B-DNA).

The two XRE-type HTH domains containing 5 α-helices (PROSITE documentation PDOC50943) interact with each other through loop α3-α4 and helix α5 whereas their two 32 Å-apart α3 helices perfectly accommodate in the successive DNA major groove of the two half-sites of the pseudopalindromic dsDNA fragment (Fig 1A). H-bonds are made by two arginine residues from α3: R35 and R39. The HTH from chain A interacts with A7’ and G6’ *via* R35 and with G4’ via R39. The HTH from chain B interacts with G6 *via* R35 and with A5 and G4 *via* R39 (Fig 1B). The interacting base pairs GC6, AT5, CG4 and AT3 of the ComR-box were previously shown to be essential for *S*. *thermophilus* ComR binding and form a highly conserved GACA motif in ComR-boxes of streptococci [30, 34]. Interestingly, R35 and R39 are strictly conserved among ComR orthologues (S1 Fig), which suggests similar ComR-DNA interactions in other streptococcal species. Additional non-specific interactions involving the phosphate-sugar backbone of DNA stabilize the ComR-DNA complex. In particular, a third arginine residue, R51 from α4, interacts with the 5’ phosphate of the G4’ nucleotide (Fig 1B).

The two TPR domains form a tight dimer characterized by an interface area of 978.5 Å^2^ and a calculated solvation free energy gain Δ^i^G of -6.2 kcal mol^-1^. This ComR dimer is stabilized by numerous interactions and considered as a biologically significant assembly by the PDBePISA [45] interactive tool used for this analysis. In particular, salt bridges are formed between two pairs of interacting residues, K87-D200 and K246-E282 (Fig 1C). Both TPR domains form a classic super-helix with a similar pitch and superimpose very well with an rmsd of 0.61 Å over 231 aligned Cα atoms. Each TPR domain is composed of 11 α-helices forming 5 TPR motifs followed by an additional C-terminal α-helix α16 called CAP. Interestingly, the TPR-2 motif (helices α8-α9) does not display the canonical pair of antiparallel α-helices. Helix α9 is broken and almost perpendicular to α8 (Fig 1A).

Interestingly, the two DNA-binding domains display distinct relative orientations with their respective peptide-binding domain (Fig 1D). This most probably results from the flexibility of loop α5-α6 linking the HTH and TPR domains. The observed asymmetry thus reflects crystallographic constraints on the flexible protein. As a consequence, the observed salt bridge between the HTH residue R58 from chain B and the TPR-2 residue E117 from chain A (Fig 1C) is most probably a crystal artifact. The intrinsic flexibility of the protein allows for the reorientation of the HTH domains and formation of a domain swapped dimer compatible with DNA binding (Fig 1A).

**Fig 2:**
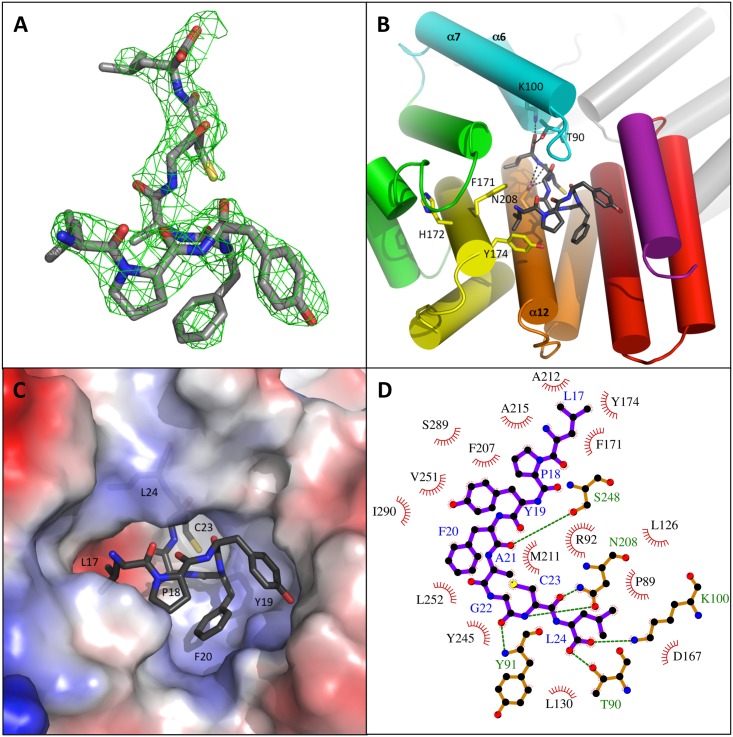
Peptide binding mode. **A–Electron density map of the bound peptide.** The Fo-Fc map, calculated by a simulated annealing protocol without peptide in the structure, is shown contoured at 1σ as a green grid with the peptide in sticks colored by atom type. **B–Peptide binding mode.** The protein is shown as cartoon colored by spectrum as chain B in Fig 1A. The bound peptide is shown as sticks colored by atom type. Residues K100, T90 and N208 H-bonding with the bound peptide are highlighted in sticks as well as residues F171, H172 and Y174. **C–Peptide-binding pocket.** ComR is shown as electrostatic surface colored by potential from blue (positive) to red (negative). The bound XIP is shown as sticks colored by atom type and its residues are labeled. **D–Schematic drawing of the ComR-XIP interaction**. The XIP peptide is shown as purple sticks with blue labels. The polar ComR residues directly interacting with XIP are shown as orange sticks with green labels. Hydrogen bonds are highlighted by green dashed lines. The hydrophobic ComR residues forming the peptide binding pocket are shown as red arcs with spokes radiating towards the ligand. The figure has been generated using Ligplot [47].

**Fig 3:**
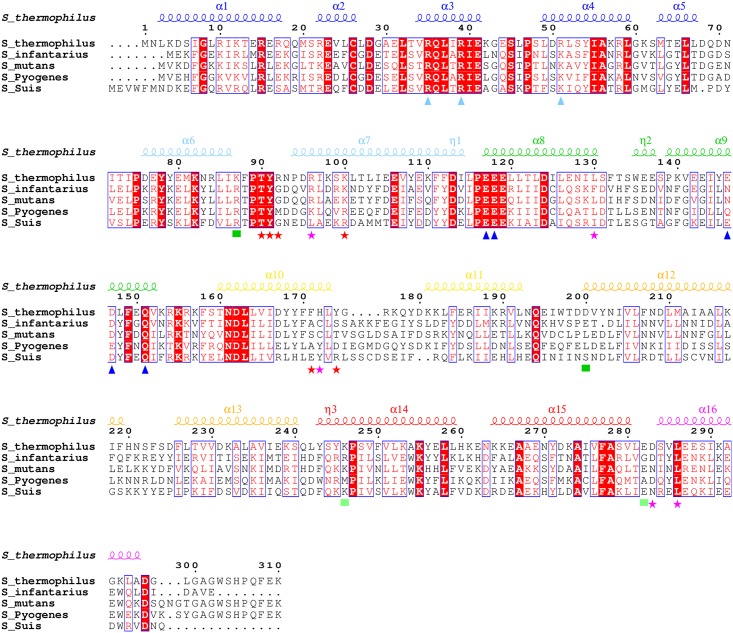
Sequence alignment of representative ComR orthologues. The ComR apo secondary structure elements of apo ComR are indicated at the top of the alignment. The α-helices are coloured according to Fig 1A. Strictly conserved residues are highlighted with red background and white labels. Conserved residues are written in red in blue frames. The similarity score has been calculated using the Rissler matrix [48]. Functionally important residues are highlighted with distinct signs at the bottom of the alignment: cyan triangles for DNA binding and HTH sequestration (R35, R39 and R51); blue triangles for HTH sequestration (E117, E118, E146, D147); green squares for dimerization (R87, D200, K246, E282); red stars for mutated residues (T90, Y91, R92, K100, F171, Y174) and pink stars for other discussed residues (R96, L130, H172, D283, L286). The sequence alignment initially generated by Clustal Ω [49] displays ComR from *S*. *thermophilus* LMD-9 (GenBank ABJ65625.1) and 4 homologues (UniProt numbers) that were experimentally validated as functional for competence activation: *S*. *infantarius* (H6PCI7), *S*. *mutans* (Q8DWI6), *S*. *pyogenes* (Q9A1Y2), *S*. *suis* (A4VSD6). The formatting of the multiple alignment has been generated using Espript [50].

In both ComR subunits, the bound XIP peptide is well defined in the electronic density (Fig 2A). An overall interface of about 775 Å^2^ and a calculated solvation free energy gain Δ^i^G of -15 kcal.mol^-1^ characterize this interaction. The hydrophobic peptide is lying on helix α12 which forms the bottom of the binding pocket. Helix α12 carries the asparagine residue N208 that H-bonds with the peptide backbone (Fig 2B), as usual for TPR domains [46]. The N-terminus of the peptide points toward the solvent whereas the C-terminus of the peptide is buried in a deep pocket (Fig 2C), characterized by numerous hydrophobic interactions (Fig 2D). The C-terminal carboxylate group forms a salt bridge with the side chain of residue K100 from helix α7 and H-bonds with T90 (Fig 2B) from the highly conserved PTY motif of loop α6-α7 (S1 Fig and Fig 3). The latter closes the deep peptide-binding pocket.

### Apo ComR is a monomer with sequestered HTH domains

**Fig 4:**
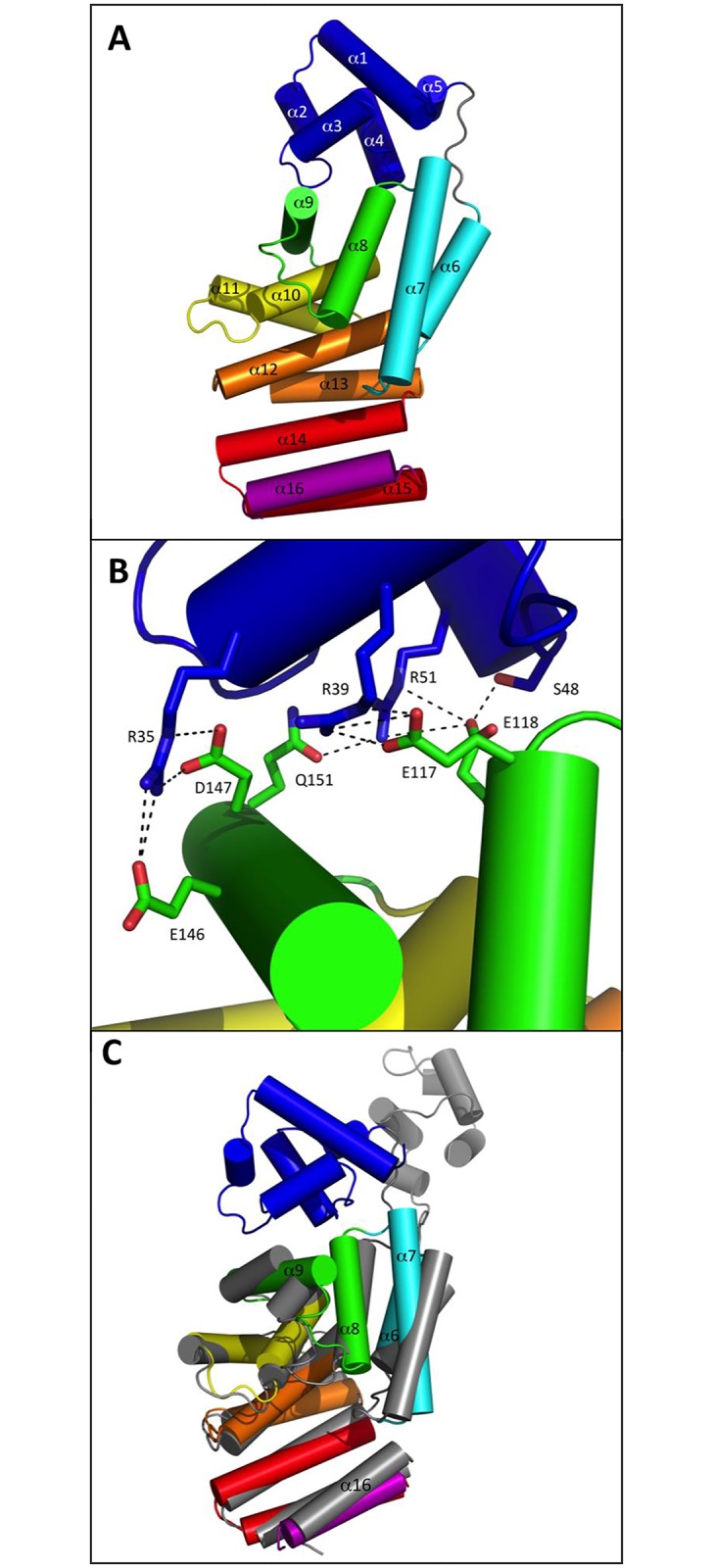
Apo conformation of ComR. **A–Overall view of the monomeric apo state.** ComR is shown as cartoon colored by spectrum from TPR-1 in cyan to TPR-5 in red with the additional CAP helix α16 in purple and the HTH-domain in blue. The α-helices are labeled. **B–Close view of the HTH sequestration mode.** The protein is shown as cartoon colored by spectrum as in panel A with the contact residues highlighted in sticks, colored by atom type and labeled. **C–Superimposition of the free and bound forms of the TPR domain.** Superimposition of the apo structure of ComR, colored by spectrum, and chain B from the ComR/XIP/DNA dimer, shown in gray.

The crystal structure of the apo form of ComR from *S*. *thermophilus* has been determined at a resolution of 1.95 Å in the space group C 2 2 2 with 1 molecule per asymmetric unit (Fig 4A). All atoms are well defined in the electron density map except for the initiating N-terminal methionine and the 11 residues of the C-terminal Strep-tag. Assembly analysis of the crystal contacts using PDBePISA [45] suggested that apo ComR is a monomer. Interestingly, crystal contacts generate a dimerization interface similar to the dimeric bound form of the protein, with a conserved K246-E282 salt bridge (S2 Fig). This suggests that this interaction is most likely the anchoring point for the formation of the dimer stabilized by the conformational change induced upon peptide binding. However, the small overall interface (537.9 Å^2^) and the poor calculated solvation free energy gain (Δ^i^G of -1.9 kcal.mol^-1^) characterizing this contact in the apo structure suggest that the monomer-dimer equilibrium is shifted toward the monomeric form in the absence of peptide and that the dimer would not be stable in the absence of crystal packing forces.

The most striking difference between the apo and peptide-bound forms of ComR concerns the relative positioning of the TPR and HTH domains. In the absence of peptide, the HTH domain is sequestered by the TPR domain of the ComR monomer, generating a new interface of 874.9 Å^2^. Interestingly, this interface involves the HTH residues R35, R39 and R51, which have been shown to be directly implicated in DNA-binding (Fig 1B). In the apo form of the protein, they form salt bridges with TPR-2 residues: R35 with E146 and D147, R39 with E117, and R51 with E118. In addition, E118 H-bonds with S48, and R51 H-bonds with Q151 (Fig 4B). The residue pair E117-E118, which belongs to the strongly conserved L_115_PEEE_119_ motif located in loop α7-α8 between TPR-1 and TPR-2 (S1 Fig and Fig 3), thus plays an important role in stabilizing the inactive apo form of the protein. As shown in Fig 3, residue Q151 of helix α9 is also strictly conserved whereas similar residues E, D, N or Q are observed at positions E146-D147 of the aligned sequences, suggesting that these residues also play an important role.

Superimposition of the apo and bound forms of the ComR TPR domain (rmsd of 2.4 Å over 212 aligned Cα atoms) (Fig 4C) showed that the main difference concerns helix α9, which was broken in the dimeric complex and forms a more canonical TPR-2 motif in the apo structure. In addition, both TPR-1 (α6-α7) and TPR-2 (α8-α9) are shifted toward the CAP helix α16 in the ternary complex, resulting in a slightly more compact conformation of the TPR superhelix, compared to the apo form. Given these observations, we then attempted to validate our proposed HTH sequestration mechanism and to understand how the peptide-induced conformational change could result in HTH release, dimerization and DNA binding.

### Peptide-binding induces dimerization and release of the sequestered HTH domains

**Fig 5:**
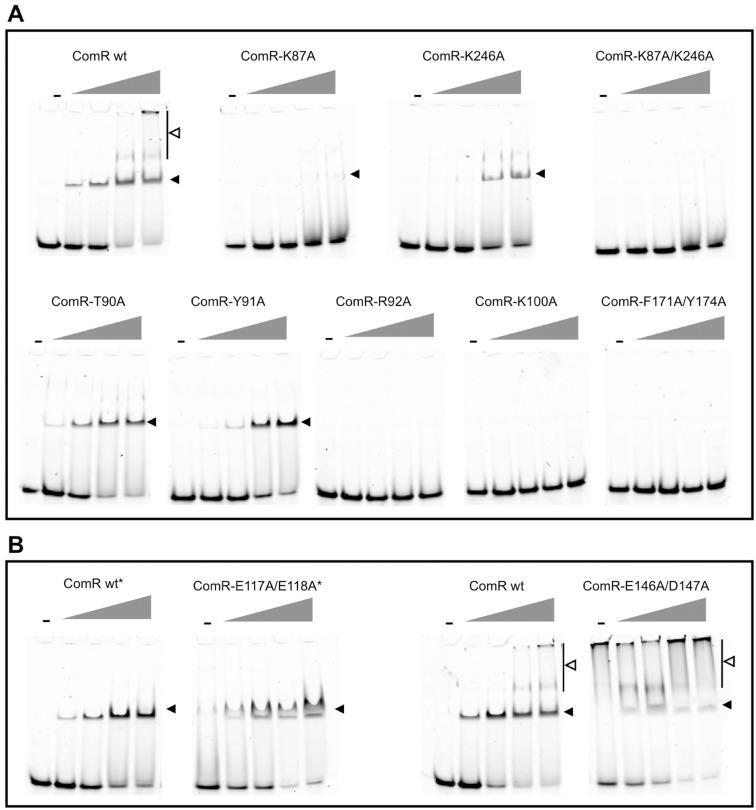
DNA binding activity. **A–EMSAs performed with ComR mutants affected in dimerization, XIP binding, and activation mechanism** (K87A, K246A, K87A/K246A, T90A, Y91A, R92A, K100A, and F171A/Y174A). **B–EMSAs performed with ComR mutants affected in HTH sequestration** (E117A/E118A and E146A/D147A). EMSAs reactions contain a 40-bp dsDNA encompassing the ComR-box (Cy3-boxP_*ster_1655*_ wt; 40 ng) and either WT (control) or mutant ComR proteins. Reactions were incubated in the absence (-) or presence of XIP. ComR was used at final concentration of 4 μM, except for mutant ComR(E117A/E118A) where a maximum concentration of 3.5 μM could be reached (*). The gray triangles represent increasing XIP concentrations: 0.2, 0.4, 0.8 and 1.2 μM. The well-defined ComRS-DNA complex and multimeric complexes are respectively indicated by closed and opened arrow heads. One representative experiment of at least 2 independent replicates is shown.

**Fig 6:**
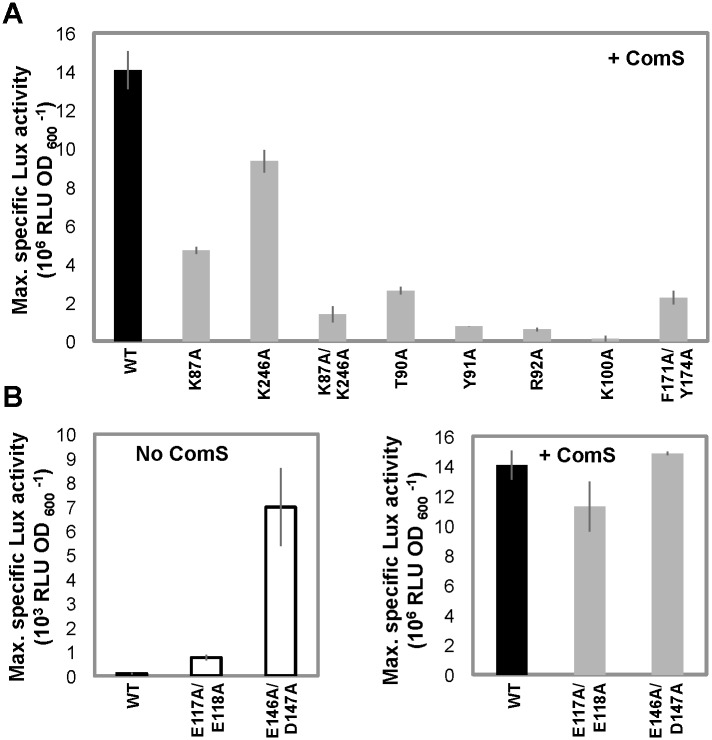
*In vivo* transcriptional activity. **A–*In vivo* activities of ComR mutants affected in dimerization, XIP binding and activation mechanism.** Maximum specific luciferase activity (RLU OD_600_^-1^) emitted by the ComS^-^ reporter strain producing ComR WT (black bar) and ComR mutants K87A, D246A, K87A/D246A, T90A, Y91A, R92A, K100A, F171A/Y174A (gray bars) grown in presence of 50 nM XIP. **B–*In vivo* activities of ComR mutants affected in HTH sequestration.** Maximum specific luciferase activity of the ComS^-^ reporter strain producing ComR WT (black bars) and ComR mutants E117A/E118A and E146A/D147A grown without (white bars) or with 50 nM XIP (gray bars). Bars represent the average of three independent repeats ± standard deviation.

**Fig 7:**
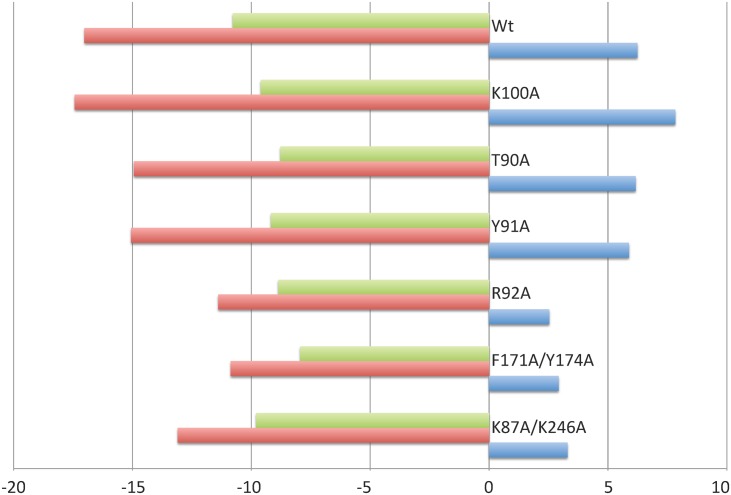
Thermodynamic signatures of XIP binding on ComR WT and mutant proteins. The enthalpy change (ΔH), the entropic contribution (-TΔS) and the Gibbs free energy change (ΔG) are shown in blue, green and red, respectively, and expressed in kcal mol^-1^.

To go further in the proposed activation mechanism, we investigated if dimerization could occur prior to DNA-binding. Size Exclusion Chromatography combined to Multi Angle Laser Light Scattering (SEC-MALLS) analysis clearly established that apo ComR is a monomer in solution, with a measured molecular weight of 41 kDa for a calculated value of 36.5 kDa (S3A Fig). Addition of XIP induced a clear shift of the elution profile and the measured molecular weight increased to 85 kDa, in agreement with the formation of a dimer (S3B Fig). However, in most SEC-MALLS experiments performed in the presence of peptide, multimers formed in a concentration-dependent manner, suggesting non-specific aggregation of the ComR dimer *in vitro*. Next, we evaluated the importance of the peptide-induced TPR dimerization in the activation process. For this assay, we engineered simple and double ComR mutants (K87A and/or K246A) to impair the formation of the K87-D200 and/or K246-E282 salt bridges stabilizing the TPR dimerization interface (Fig 1C). SEC-MALLS analysis of the double mutant K87A/K246A demonstrated that addition of peptide induced the formation of very high molecular weight aggregates, suggesting that the peptide-bound form definitely needs to dimerize through this interface to be stable. On the other hand, electrophoretic mobility shift assays (EMSA) revealed that the binding activity of the single point mutants K87A and K246A is partially decreased, while the double mutant K87A/K246A is unable to bind DNA, even at high concentrations of peptide (Fig 5A). *In vivo* luciferase assays further demonstrated that addition of XIP partially induced the reporter fusion for the single amino-acid substituted ComR mutants K87A and K246A while the double mutant K87A/K246A was almost inactive (Fig 6A). It is also interesting to note that the K87A point mutant showed less activity than K246A, suggesting that the K87-D200 salt bridge, which is only observed in the ternary complex, has a predominant role in ComR oligomerization. Finally, Isothermal Titration Calorimetry (ITC) measurements (Table 1, Fig 7) showed that the K87A/K246A mutations impairing dimerization did not affect the affinity of ComR for the XIP peptide. Taken together, these results demonstrate that peptide-induced TPR dimerization is required for DNA binding.

**Table 1:** XIP binding on ComR WT and ComR mutant proteins as measured by ITC.

ComR	stoichiometry	K_D_ (μM)
WT	0.8	0.010 ± 0.001
K100A	0.8	1.175 ± 0.245
T90A	0.7	0.244 ± 0.108
Y91A	0.9	0.141 ± 0.033
R92A	1.2	0.277 ± 0.023
F171A/Y174A	1.1	0.087 ± 0.009
K87A/K246A	0.8	0.009 ± 0.003

In order to confirm that the peptide activation mechanism is based on the release of the sequestered HTH domains, we mutated the TPR-2 residues that form salt bridges with the HTH domain. Specifically, we created the double mutants ComR(E117A/E118A) and ComR(E146A/D147A) in order to disrupt the interactions with residues R35, R39, and R51 involved in DNA binding (Fig 4B). *In vivo* luciferase assays (Fig 6B, left panel) revealed that the two double mutants are constitutively active compared to wild-type ComR in absence of XIP (~10- and ~70-fold increase, respectively) and remain similarly active compared to the wild type in presence of XIP (Fig 6B, right panel). Additionally, the DNA-binding activity of both mutated proteins in the absence of XIP was confirmed by EMSA experiments (Fig 5B). A strong interaction was observed in the absence of XIP for the mutant ComR(E146A/D147A), resulting in the formation of multimeric complexes similar to what was observed for ComR WT in the presence of high XIP concentrations (Fig 5B, right panels). In contrast, less binding was observed for ComR(E117A/E118A) in our assay (Fig 5B, left panels). This suggests that the (E146A/D147A) mutation more efficiently impair the inactivating HTH sequestration than the (E117A/E118A) mutation. This might be explained by the fact that E146 is the position where the break is observed in the active conformation of helix α9 (Fig 4). The (E146A/D147A) mutation could thus help stabilize the broken conformation of helix α9. In addition, the activity of the double mutant E117A/E118A confirms that the E117-R58 salt bridge observed in the asymmetric ternary complex is not required for the activity but most probably results from crystallographic packing constraints. Taken together, these results strongly suggest that peptide binding induces a conformational change, which introduces a break in helix α9 resulting in the release of the sequestered HTH domains and dimerization.

### Identification of key ComR residues for XIP binding and activation

In order to gain insights into the detailed molecular mechanism of this activation process, we mutated ComR residues involved either in peptide binding or in stabilization of the active conformation.

We first tested if the interactions made with the C-terminal carboxyl group of the peptide are important for binding and/or activation. The K100A mutation, which disrupts the salt bridge with the XIP carboxylate, resulted in a drastic loss of affinity, with a K_D_ value of 1.2 μM compared to the value of 10 nM measured with the wild-type protein. The T90A mutation, which disrupts a H-bond with the peptide, displayed an intermediate behavior with a K_D_ value of 240 nM (Table 1). In addition, EMSA and *in vivo* experiments clearly demonstrated that both DNA-binding (Fig 5A) and transcriptional activities (Fig 6A) are proportional to the affinity of both mutants T90A and K100A for the peptide. As the K100A mutant is dramatically affected, these results demonstrate that residue K100 plays an essential role in peptide binding.

We then investigated more globally the highly conserved PTY motif of loop α6-α7, which includes residue T90. Comparison of the two crystal structures revealed that, upon peptide binding, TPR-1 is shifted toward the CAP helix α16, bringing the side chain of Y91 in stacking interaction with the conserved residue L286. Concomitantly, residue R96 forms a salt bridge with D283, thus further stabilizing the TPR superhelix in the compact conformation observed in the dimeric complex (Fig 4C). Interestingly, loop α6-α7 carrying the conserved PTY motif also carries residue K87. The peptide-induced conformational change brings K87 in proximity to residue D200 from the neighboring subunit with which it forms a salt bridge stabilizing the active dimer. We also demonstrated that mutating residue Y91, whose side chain does not directly interact with the bound peptide, resulted in a XIP K_D_ value of the same order of magnitude than observed with T90A mutant (Table 1). Furthermore, the Y91A mutant also displayed a reduced DNA-binding capacity (Fig 5A) and a drastic drop in its transcriptional activity (Fig 6A). Taken together, these results thus suggest that loop α6-α7 carrying the conserved PTY motif is directly involved in the activation mechanism. Moreover, residue R92, which forms hydrophobic interactions with the peptide, H-bonds with the backbone carbonyl of residue L130, the C-terminal residue of helix α8 of TPR-2 (Fig 4C). As observed with the Y91A mutant, the R92A mutation resulted in a decreased affinity for XIP (Table 1). In addition, the R92A mutant displayed a drastic loss in both its DNA-binding capacity and its transcriptional activity (Figs 5A and 6A). The role of this residue could be to induce the conformational change of the flexible loop α8-α9, resulting in the break of helix α9 (Fig 4C). This observation also reinforces the role of loop α6-α7 from TPR-1 in stabilizing the closed active conformation of the TPR domain.

Finally, the conformational change observed in TPR-2 upon peptide binding induces a slight reorientation of helix α10 of TPR-3, resulting in a new interaction network for His172 and a reorientation of residues F171 and Y174, which form a hydrophobic pocket where the N-terminal Leucine residue of the XIP octapeptide binds (Fig 2B). Interestingly, the F171A/Y174A double mutant displayed a drastic loss in its DNA binding activity (Fig 5A) and a strongly decreased *in vivo* activity (Fig 6A). This demonstrates that residues F171 and Y174 play an essential role in the ComR activation mechanism. On the other hand, and in agreement with our previous study demonstrating that the heptapeptide ComS_18-24_ lacking this N-terminal Leucine is also fully active [29], the affinity for ComS_17-24_ was almost unchanged by the F171A/Y174A mutation, with a K_D_ value of about 87 nM (Table 1). Given this, the exact role of these residues remains unclear. Analysis of the thermodynamic signatures of the interactions between the XIP peptide and ComR mutant proteins (Fig 7), demonstrated that all these interactions are entropy driven, in agreement with the peptide binding mode displaying a substantial non-electrostatic contribution (Fig 2D).

### Identification of important XIP residues for binding and activation

**Fig 8:**
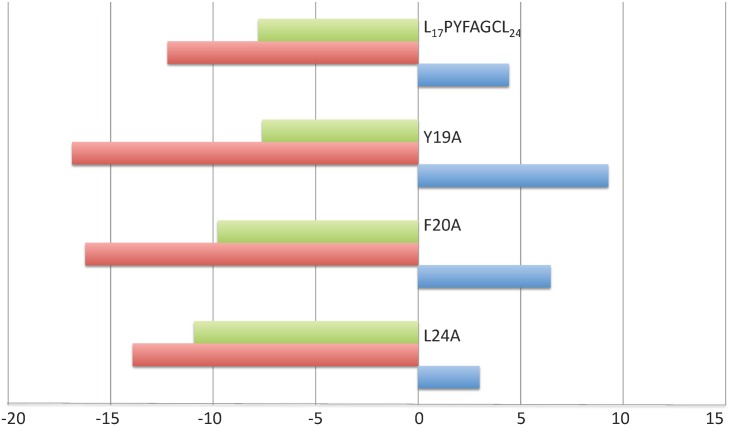
Thermodynamic signatures of the binding on ComR of XIP WT and variants. The enthalpy change (ΔH), the entropic contribution (-TΔS) and the Gibbs free energy change (ΔG) are shown in blue, green and red, respectively, and expressed in kcal mol^-1^.

A previous analysis of XIP variants in which each residue had been mutated into alanine had already demonstrated that only substitutions concerning the XIP residues Y19, F20 or L24 drastically reduced the *in vivo* activation efficiency of the peptide [30]. In order to go further in the understanding of the activation mechanism, we compared the affinity of ComR for these Y19A, F20A and L24A XIP variants with a reference **KD** value of 8 nM measured with the wild-type XIP octapeptide (Table 2). Our ITC experiments demonstrated that the inactivating substitutions F20A or L24A drastically increased the K_D_ value to 2 μM and 1.5 μM, respectively, whereas the Y19A inactivating substitution had only little impact on the affinity of the protein, with a K_D_ value of 52 nM. Taken together, these results thus suggest that XIP(Y19) plays an essential role in the activation mechanism of the ComRS system. Analysis of the thermodynamic signatures of the interactions between these XIP variants and ComR WT demonstrated that the substitutions did not affect the energy pattern of the interaction, which remains entropy driven in all cases (Fig 8).

**Table 2:** Binding on ComR of WT and variants XIPs as measured by ITC.

Peptide	stoichiometry	K_D_ (μM)
WT (L_17_PYFAGCL_24_)	1.1	0.008 ± 0.004
Y19A	1.5	0.052 ± 0.013
F20A	1.2	2.127 ± 0.226
L24A	1.75	1.552 ± 0.332

## Discussion

**Fig 9:**
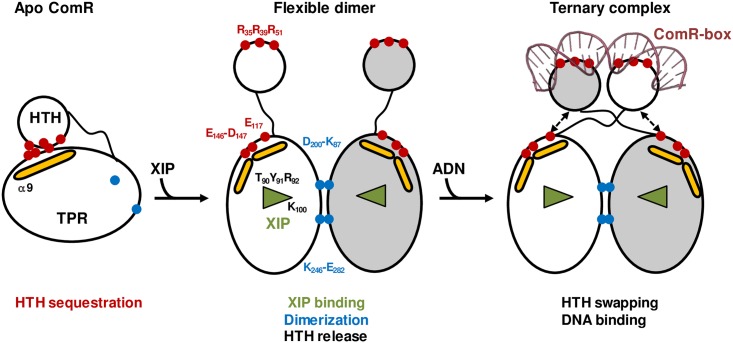
Mechanism of ComR activation by the XIP peptide. The empty XIP-binding pocket of the apo form of ComR is represented by an open triangle. Helix α9, which undergoes a major conformational change, is shown as a yellow stick. Key ComR residues are labeled, in black for XIP binding and activation residues (T90, Y91, R92, and K100), in red for residues involved in DNA binding and HTH sequestration (R35, R39, R51, E117, D146, and E147) and in blue for residues of the dimerization interface (K87, D200, K246, and E282).

This structure-function analysis has allowed us to elucidate the molecular activation mechanism of the ComRS system from *S*. *thermophilus*. In the absence of XIP, the mature form of the signaling peptide ComS, the transcription regulator ComR is mainly in a monomeric inactive form characterized by the sequestration of the HTH DNA-binding domain by the TPR domain. When the latter binds an activating peptide, it is shifted toward a closed conformation, inducing the release of the HTH domains and dimerization. The identification of essential residues of both ComR and XIP allowed us to propose a detailed activation mechanism (Fig 9).

The C-terminal carboxyl group of the bound peptide forms a salt bridge with K100 and H-bonds with T90. This essential interaction induces a shift of TPR-1, and in particular of loop α6-α7, to a closed conformation burying the peptide in the core of the protein. In this conformation of loop α6-α7, residue R92 interacts with the C-terminal carbonyl group of helix α8 and allows the induced conformational change to propagate to TPR-2. A slight reorientation of helix α8, which carries residues E117 and E118 at its N-terminus, most likely destabilizes the salt bridges with the HTH residue R39. Most importantly, loop α8-α9 undergoes a drastic conformational change, resulting in a break of helix α9 at residue E146. As a consequence, the salt bridges formed by residues E146-D147 and the HTH residue R35 are disrupted, thus releasing the sequestered HTH domain. The drastic conformational change occurring in TPR-2 probably involves the adjacent helix α10 carrying residues F171 and Y174, which have also been shown to play an essential role in the activation mechanism. It further results in new interactions between loop α6-α7 and the C-terminal helix α16. In addition, residue Y91 makes hydrophobic interaction with L286, and R96 forms a salt bridge with D283. Helix α16 is thus shifted toward the peptide and forms specific hydrophobic interactions with residue Y19 from its cognate XIP peptide L_17_PYFAGCL_24_. In this compact conformation, which closes the peptide-binding pocket, residues K87 and D200 are properly orientated to form a salt bridge between two neighboring molecules, thus stabilizing the active ComR dimer.

Interestingly, sequence alignment analysis (S1 Fig and Fig 3) revealed that residues shown to be essential in the activation mechanism of ComR from *S*. *thermophilus* are conserved in most orthologues, suggesting a common molecular mechanism despite distinct specificities toward their cognate type-II or type-III signaling peptides. This hypothesis is supported by the crystal structure of ComR from *S*. *suis* reported in the joint publication of Shanker et al. [51], which displays a monomeric conformation similar to the apo form of ComR from *S*. *thermophilus*, with a sequestered HTH domain. Furthermore, a weak dimer similar to that observed in the crystal structure of the apo form of ComR from *S*. *thermophilus* is also present in the structure from *S*. *suis*, suggesting a conserved dimerization mode upon peptide binding. The latter displays however a dimer configuration of the TPR domains resembling the peptide-bound state of ComR from S. *thermophilus*. As a consequence, the interactions between TPR-2 and the HTH domain are not as strong as observed in *S*. *thermophilus*. Only one salt bridge is observed between residues R38 and E115, which is equivalent to the R39/E117 interaction from *S*. *thermophilus*. This might explain the distinct thermodynamic behaviors observed in the ITC experiments. Additionally, this discrepancy and other small structural differences observed between the inactive ComR structures could also reflect different permissiveness toward ligand binding [51].

ComR from *S*. *thermophilus* has been shown to be permissive [30]. In particular, it is more efficiently activated by the type II peptide MGLDWWSL from *S*. *mutans* than by the type I peptide VPFFMIYY from *S*. *vestibularis*. It is also activated by the casein-derived octapeptide GAWYYVPL. The hydrophobic pockets occupied by the essential residues F20 and L24 from the cognate XIP peptide L_17_PYFAGCL_24_ are wide enough to accommodate larger residues like W and Y respectively. In contrary, if these pockets are not occupied, the affinity of the peptide decreases drastically. This is the case for the F20A and L24A XIP variants. Interestingly, the casein-derivative peptide LAYFYPEL, which contains residues equivalent to XIP F20 and L24 is inactive. This is most probably due to the substitution of G23 by a P, impairing the peptide to adopt a conformation suitable to fit in the ComR binding site. The XIP residue Y19, which in contrary to F20 and L24 is not essential for the affinity of the interaction, has been shown to play an essential role in the activating conformational change by recruiting helix α16 *via* hydrophobic interactions with residues S289 and I290. Interestingly, it can be replaced by other aromatic residues without loss of activity [30]. However, activating peptides with highly divergent sequences can adopt distinct conformations and their exact binding mode is difficult to predict.

The presence in the peptide-binding site of ComR from *S*. *suis* of the N-terminal residues of a symmetry-related molecule of the crystal packing gives also some clues on the peptide recognition mode. In particular, it suggests that a potential activating pheromone peptide is first both recognized and selected by the apo-pocket before the activating conformational change occurs (joint publication, [51]). The bottom of the peptide-binding pocket formed by helix α12 corresponds to the variable face described in the accompanying paper [51] and can accommodate different peptide sequences, ensuring the selectivity of the peptide recognition. In addition, comparison of the peptide binding sites of ComR from *S*. *thermophilus* and *S*. *suis* shows that the wide hydropobic pocket occupied by the C-terminal XIP L24 in *S*. *thermophilus* is partially filled by the side chain R167 in ComR from *S*. *suis*. Only a small range of residues can fit in this reduced pocket, explaining the higher selectivity of ComR from *S*. *suis*, as presented in the accompanying paper [51].

The asymmetric structure observed in the ComR/XIP/DNA ternary complex of *S*. *thermophilus* suggests that once the HTH-TPR interaction is broken, the linker loop would remain flexible resulting in various relative orientations of the HTH and TPR domains. The domain-swapped dimeric form of the protein is most probably stabilized by the interaction of the HTH domains with the two half sites of the pseudopalindromic ComR-box of the targeted promoter. This dimerization mode is reminiscent to the dimeric structures of Rgg, PrgX and PlcR. However, in these other RNPP proteins the swapped dimer is observed in the apo form as well as in the presence of bound peptide. In particular, the apo form of Rgg2 from *S*. *dysgalactiae* displays a dimeric structure in which the swapped HTH domains are linked by a disulfide bridge [27]. An activation mechanism has been proposed, involving the formation of a small β-sheet between the bound linear SHP-peptide and the flexible C-terminal tail of the protein, as observed in PrgX from *Enterococcus faecalis*. However, in the PrgX repressor, this C-terminal region is involved in the stabilization of a head-to-head interaction between two PrgX dimers forming a tetramer required for binding with a 70-bp DNA loop encompassing two operator sites, i.e. *prgX* and *prgQ* [13]. Interaction of PrgX with the cCF10 pheromone has been shown to destabilize the PrgX tetramer, effectively decreasing the affinity of the repressor toward the *prgQ* operator [13]. In the well-characterized PlcR-PapR system of *Bacillus cereus*, peptide binding destabilizes the preformed PlcR dimer, resulting in the loss of interactions between the long α-helices that link TPR and HTH domains from both subunits. In the presence of DNA, this gain of flexibility allows the linker helices to break in two individual helices for proper positioning of the HTH domains in the major groove of the two half-sites of the DNA binding sequence [15]. Finally, binding of the NprX pheromone to NprR has been shown to stabilize a tetrameric conformation without similarity with the subunit interaction modes of the other RNPP transcriptional regulators [20]. Interestingly, NprR is a bifunctional quorum sensor, acting as a Rap-like phosphatase in the absence of peptide [21]. The apo form of NprR inhibits sporulation by binding to the phosphotransferase Spo0F, in the same way as observed with RapH [16]. The N-terminal region of the TPR domain of NprR and Rap contains a highly flexible extension, which forms two additional TPR motifs in the presence of peptide but displays a 3-helix bundle conformation in the presence of the target protein, i.e. Spo0F for NprR and RapH. Thus, despite sharing similar folds and peptide binding modes, RNPP proteins display distinct molecular regulatory mechanisms.

Remarkably, the HTH sequestration mechanism does not seem to be conserved in Rgg [27], further demonstrating that ComR is not an Rgg-like protein as initially reported [22, 29]. We thus propose that ComR is a new member of the RNPP family, without particular similarity with Rgg. Concerning the biological relevance of this drastic control mechanism, it is important to recall that competence is highly regulated in streptococci [35]. It is a well-described transitory process, which is not only energetically expensive but could also disturb cell division and chromosome integrity when unregulated [35]. Based on this, it appears that in the absence of the inducing peptide, the HTH sequestration mechanism observed in the apo form of ComR ensures that the regulator is completely locked and unable to activate DNA transformation. Such a sequestration mechanism has already been reported in the light-regulated LOV-HTH DNA-binding protein EL222 [52]. However, to the best of our knowledge, such a peptide-mediated strict control of a transcriptional regulator by the sequestration of its DNA-binding domain has never been reported so far.

To conclude, our results not only allowed us to propose a detailed molecular mechanism of the ComR activation process but also offer opportunities to engineer, for instance, well- controlled peptide-based expression systems in streptococci and other Gram-positive bacteria. In addition, they give interesting insights into the specificity of the peptide recognition, which could be useful for the future rational design of molecules specifically inhibiting competence in pathogenic streptococcal strains. This therapeutic approach would help decrease the fitness of the bacteria during infection and limit the acquisition of pathogenic islands or antibiotic resistance encoding genes. Particularly, a first approach could be the identification of high-affinity peptide analogues that could compete with XIP binding without productive ComR activation. Indeed, our ITC experiments and the structure of ComR from *S*. *suis* [51] show that the peptide-binding pocket of ComR can accommodate XIP variants that bind in a non-productive manner and could serve as basis for the rational design of such inhibitors.

## Materials and Methods

### Bacterial strains and growth conditions

The bacterial strains used in the functional analysis of ComR are listed in S1 Table. *Escherichia coli* was generally grown in LB medium with shaking at 37°C [53]. *S*. *thermophilus* was grown at 37°C in M17 broth (Difco Laboratories Inc., MI) or in CDM [54]. All synthetic media contained 1% (wt vol^-1^) lactose (M17L and CDML broth, respectively). When required, ampicillin (200 μg ml^−1^ for *E*. *coli*), streptomycin (50 μg ml^−1^ for *E*. *coli*), or chloramphenicol (5 μg ml^−1^ for *S*. *thermophilus*) was added to the media. Plates inoculated with *S*. *thermophilus* cells were incubated anaerobically (BBL GasPak systems, Becton Dickinson, NJ) at 37°C.

### DNA techniques and electrotransformation

*E*. *coli* strain TOP10 (Invitrogen, CA) was used as a host in all cloning experiments, and all DNA manipulations were performed according to standard procedures described in [53]. Electrotransformation of *E*. *coli* was performed as described previously [55]. PCRs were performed with Phusion high-fidelity DNA polymerase (Finnzymes, Espoo, Finland). The primers used in this study were purchased from Eurogentec (Seraing, Belgium) and are listed in S2 Table.

### Construction of mutant *comR-strep* expression plasmids

Plasmids used in this study are listed in S3 Table. The mutant *comR*_LMD-9-_ *strep* expression plasmids were obtained by replacing the desired codon(s) in the WT *comR*_LMD-9-_ *strep* open reading frame (ORF), which is carried on plasmid pBADcomR_LMD-9_-strep [30], by the alanine-encoding codon GCT. The desired mutation was introduced in the template vector either *in vitro* by Mutagenex Inc. (Suwanee, GA) (in case of pBADcomR_E117A,E118A_-strep, pBADcomR_E146A,D147A_-strep, pBADcomR_Y91A_-strep, pBADcomR_K100A_-strep and pBADcomR_F171A,Y174A_-strep) or by inverse PCR using partially overlapping primers (in case of plasmids pBADcomR_T90A_-strep, pBADcomR_R92A_-strep, pBADcomR_K87A_-strep, pBADcomR_K246A_-strep, and pBADcomR_K87A,K246A_-strep). In the latter case, the obtained PCR product was re-circularized by Gibson method [56], and the template carrying the WT *comR*_LMD-9_-*strep* gene was eliminated by *Dpn*I digestion. Plasmid pBADcomR_K87A,K246A_-strep which bears two mutations was created by performing two successive mutagenesis rounds.

### Construction of mutant strains of *S*. *thermophilus* LMD-9 by natural transformation experiments

The activity of ComR mutants was studied *in vivo* in a ComS^-^ LMD-9 derivative strain that bears a P_*comS*_ *-luxAB* reporter fusion at the *blp* locus [30]. Introduction of the desired mutation in *comR* and replacement of *comS* by the P_*32*_ *-cat* cassette in the reporter strain LF121 was performed in one step by double homologous recombination of a linear DNA fragment, which was introduced by natural transformation. The chimeric mutagenesis DNA fragment was created *in vitro* by joining three PCR fragments by overlapping PCR. The first PCR fragment included the 1-kb region located upstream of *comR* and extends up to 252 pb in the *comR* ORF. The second PCR fragment of ~0.66 kb encompassed the desired mutation in *comR*, and was amplified on the corresponding mutant *comR-strep* expression plasmid. The third PCR product was a 2.5-kb DNA fragment containing the STOP codon of *comR*, the *comS*::P_*32*_ *-cat* cassette and ~1 kb-region located downstream of *cat*, and was amplified on ComS^-^ strain LF134. The three PCR products were joined together thanks to the complementarity of the PCR fragments, and the full-length product was amplified with the external primers. Natural transformation of the purified overlapping PCR products in strain LF121 peptide was performed as previously described [29, 57]. Transformants were selected on M17 broth containing chloramphenicol. Integration of the antibiotic resistance cassette at the right location was checked by PCR and the whole transformed region was sequenced.

### Purification of ComR–StreptagII proteins

Purification of ComR-StreptagII proteins (StreptagII at C-terminus) was performed as described previously with some modifications [30, 58]. *E*. *coli* strain BL21 Gold cultures were grown in 2YT medium at 15°C until an OD_600_ of ~ 0.5, cooled down on ice for 10 minutes, and induced by addition of 0.2% of L-arabinose (wt vol^-1^) before continuous shaking at 28°C for 4 h. Cells were then harvested by centrifugation (5,000 × *g* during 15 min at 8°C). The pellets were resuspended in cold buffer W (100 mM Tris-HCl pH 8.0, 150 mM NaCl, 1 mM EDTA) supplemented with 0.5 mg ml^−1^ of lysozyme, incubated for 30 min at 4°C, and sonicated at 4°C. The soluble fractions were collected after centrifugation (15,000 × *g* for 60 min at 4°C) and purification of the recombinant ComR-StreptagII proteins was performed by one-step affinity chromatography with 1 or 2 ml Strep-Tactin Superflow column (IBA BioTAGnology, Göttingen, Germany) according to the manufacturer's instructions. For structural and biophysical analyses, the purification protocol was completed by a size exclusion chromatography step using a HiLoad 16/600 Superdex 75 prep grade column (GE Healthcare) equilibrated in buffer W plus 10% (vol vol^-1^) glycerol. Purified proteins were aliquoted, flash frozen and conserved at -20°C. Their purity was visualized by SDS-PAGE and their concentrations were measured using a Nanodrop apparatus (protein A_280_ method) (ThermoFisher Scientific).

For expression and purification of the Selenomethonine (SeMet)-labelled form of ComR, cells of an overnight culture in 2YT medium were transferred in M63 minimal medium and grown at 37°C for one day in the presence of ampicilin. Starved cells were transferred in fresh M63 medium, grown at 37°C until OD_600_ of ~1 and supplemented with 100 mg l^-1^ of an amino acid mix (Leu, Ile, Val, Trp, Phe, Lys, Ala, Arg, Asp, Gly, Asn, Pro, Ser, Thr, His, Gln, and Glu) and 62.5 mg l^-1^ of selenomethionine. Finally, expression was induced at OD_600_ of ~1.5 by adding 0.2% L-arabinose (wt vol^-1^) and the culture was incubated overnight at 15°C. The purification protocol was the same as reported above for the label-free protein.

### Peptide and DNA samples preparation

In *S*. *thermophilus* LMD-9, the C-terminal heptapeptide 18–24 from the ComS precursor (MKTLKIFVLFSLLIAILPYFAGCL) has been shown to be the minimal active form of the signaling peptide [29]. However, co-crystallization experiments were performed with the ComS_17-24_ octapeptide, which is more stable and was used in the characterization of the ComRS system [30]. The corresponding synthetic XIP peptide LPYFAGCL as well as the variants used in the functional analysis were supplied by GenScript or Peptide 2.0 (Chantilly, VA) and resuspended at a concentration of 50 mM in 50% DMSO / 50% protein storage buffer.

The ComR-box is a 20 bp pseudo-palindromic motif characterized by a conserved inner GACA/TGTC inverted repeat that is crucial for efficient binding of the ComRS complex [30]. Two lyophilized complementary oligonucleotides corresponding to the ComR-box from the *comX* promoter (5’-**T10-A9-G8-**T7-**G6-A5-C4-A3-T2-A1.T1’-A2’-T3’-G4’-T5’-C6’-**T7’-**C8’-T9’-A10’**-3’) were chemically synthesized and purified by HPLC (supplied by GenScript). They were resuspended at a final concentration of 1 mM in 10 mM Tris–HCl pH 8.0, 75 mM NaCl and 0.5 mM EDTA. Complementary strands were then equimolarly mixed and subsequently hybridized at 96°C on a Thermomixer (Eppendorf France S.A.S.) for 60 min under shaking at a constant speed of 300 rpm, and then left overnight at room temperature for slow cooling. The resulting dsDNA fragments were flash-frozen in liquid nitrogen and stored at -20°C.

### Crystallization assays

Crystallization trials were performed at 18°C using a Cartesian robot and commercial kits. Initial hits were reproduced and optimized manually using the hanging drop method and homemade solutions. The apo form of ComR crystallized in 20% PEG 8000 (wt vol^-1^), 0.2 M ammonium sulfate, 0.1 M sodium cacodylate pH 6.4 at a concentration of 4 mg ml^-1^. The ComR/XIP/DNA ternary complex was prepared with the respective final concentrations of 6.5 mg ml^-1^ (175 μM) ComR, 1 mM XIP and 175 μM of the specific 20-bp dsDNA (ComR-box of P_*comX*_) resuspended in the protein storage buffer. The complex crystallized in 30% PEG 550 MME (wt vol^-1^), 0.1 M MgCl_2_, 0.05 M Tris-HCl pH 8.5. Crystals were flash frozen in the crystallization solution supplemented with 25% glycerol and conserved in liquid nitrogen prior X-ray diffraction assays.

### X-ray diffraction data collection

Crystals of the apo form of ComR diffracted well and a native data set was collected at 1.95 Å resolution. Crystals obtained with the SeMet-labelled form of the protein diffracted at 2.8 Å resolution and were used to collect a single wavelength anomalous diffraction (SAD) data set. Both data sets were collected on beamline ID29-1 at the European Synchrotron Radiation Facility (ESRF, Grenoble, France). Crystals of the ComR/XIP/DNA complex diffracted at lower resolution. The structure was solved by using a native data set collected at 2.6 Å resolution on beamline ID23-1 at ESRF. X-ray diffraction data and refinement statistics are summarized in S4 Table.

### X-ray diffraction data processing and model refinement

All data sets were processed using the XDS package [59]. Initial phasing, sub-structure determination and model building were performed using the SAD data set of the apo form of ComR and the CRANK2 pipeline [60] of the CCP4 interface [61]. The positions of the 5 selenomethionines contained in the protein sequence were identified. The final model of apo ComR was refined at 1.95 Å resolution against the native data set using the PHENIX wizard [62] and manually optimized using COOT [63]. Atomic coordinates and structure factors of apo ComR have been deposited in the Protein Data Bank (PDB ID 5JUF).

The structure of the ComR/XIP/DNA ternary complex was solved by molecular replacement using PHASER [64] in the CCP4 [61] interface, and three search ensembles: the apo ComR TPR-domain (residues 117 to 297), the apo ComR HTH-domain (residues 1 to 69), and a 18-bp DNA fragment in the B-form. Refinement was performed using the PHENIX wizard [62] and manually optimized with COOT [63]. Atomic coordinates and structure factors of the complex have been deposited in the Protein Data Bank (PDB ID 5JUB). Data processing and refinement statistics are given in S4 Table.

### Structural analysis

We used the Protein structure comparison service PDBeFold (http://www.ebi.ac.uk/msd-srv/ssm) [65] and the Protein Interfaces, Surfaces and Assemblies service PDBePISA (http://www.ebi.ac.uk/pdbe/prot_int/pistart.html) [45] at the European Bioinformatics Institute, to compare crystal structures and analyze their assemblies, respectively. We used the PyMOL Molecular Graphics System [66] to analyze the 3D structures and prepare figures.

### Biophysical analysis

Isothermal titration calorimetry (ITC) experiments were performed at 20°C in 20 mM Tris-HCl pH 8.0, 150 mM NaCl, 1 mM EDTA with an ITC200 calorimeter (Microcal). The protein concentration in the microcalorimeter cell and the peptide concentration in the syringe were set at 50 μM and 500 μM, respectively. A first injection of 0.4 μl, which was not taken into account for the fitting, was followed by 20 injections of 2 μl at intervals of 180 s. The peptide concentration used in the calculations is based on the weight of the lyophilized peptide and is therefore inaccurate because there may be a significant quantity of bound water, and salts or counter ions. However, the calculated number of binding sites, *n*, is always close to 1, in agreement with the 1:1 ratio observed in the crystal structure of the ternary complex. This demonstrates that the peptide concentrations are properly estimated and that calculated **KD** values are reliable. The data were analyzed using the MicroCal Origin software provided by the manufacturer. The curves displayed only one transition, suggesting that peptide binding and dimerization occur concomitantly. It was therefore not possible to distinguish the two events and the fitting was performed with the one-binding-site model. The calculated **KD** are therefore apparent values.

SEC-MALLS experiments were performed using a Viskotec TDA305 triple detector array with an integrated GPC_max_ VE 2001 system (Malvern, France). The protein samples were injected at a concentration of 2 mg ml^-1^ (≈ 40 μM) in the presence of 1 mM XIP or alone at 8 mg ml^-1^ (≈ 160 μM) on an Agilent Bio-Sec 3 column (Agilent technologies) equilibrated in 20 mM Tris-HCl pH 7.5, 150 mM NaCl, 1mM EDTA. The protein sample was injected in the same buffer in the presence of 5% glycerol. The OmniSEC software of the manufacturer was used for acquisition and analysis of the data. Bovine serum albumin (Sigma–Aldrich) was used as standard reference protein for detector calibration.

### Luciferase assays

Luciferase assays were performed as previously described [30, 67]. Growth (OD_600_) and luciferase (Lux) activity (expressed in relative light unit; RLU) of the cultures were monitored after addition of 50 nM of XIP peptide during 5 h in a Varioskan Flash multi-mode reader (ThermoFisher Scientific).

### Electrophoretic mobility shift assays

Purified ComR mutants were mixed with a 40-bp dsDNA fragment carrying the ComR box of P_*ster_1655*_ coupled to the Alexa 555 fluorophore as previously described [30]. The gel shift reactions (20 μl) were performed in binding buffer (20 mM Tris-HCl pH 8.0, 150 mM NaCl, 1 mM EDTA, 10% glycerol [vol vol^-1^], 1 mg ml^−l^ BSA), and increasing XIP concentrations were added. The reactions were incubated at 37°C for 10 min and analyzed on native Mini-PROTEAN TBE Precast 4–20% Gel (Bio-Rad, Hercules, CA) in TBE buffer. DNA complexes were detected by fluorescence on the Ettan DIGE Imager with bandpass excitation and emission filters of 540/25 and 595/25 nm, respectively (GE Healthcare, Waukesha, WI).

### Accession numbers

The sequence of the ComR gene from *Streptococcus thermophilus* LMD-9 is deposited in GenBank under number ABJ65625.1

The 3D coordinates and the diffraction data of the crystal structures of ComR from *S*. *thermophilus* in the apo form and in complex with XIP and DNA have been deposited in the Protein Data Bank under IDs 5JUF and 5JUB, respectively.

## Supporting Information

S1 Fig Conservation of three key amino acids motifs in ComR orthologues. The HTH DNA binding domain (A), the PTY motif (B), and the LPEEE motif (C) are shown. Non-redundant representatives of ComR orthologues from each *Streptococcus* species (56 sequences used to generate a phylogenetic tree of ComR orthologues [35]) were aligned using BLAST on NCBI website and sequence logos were created using Weblogo (http://weblogo.berkeley.edu/logo.cgi) [68]. Bits represent the relative frequency of amino acids. X-axes refer to the position of residues in the ComR sequence of *Streptococcus thermophilus* LMD-9. Arrows highlight key residues.
(TIFF)

S2 Fig Weak apo ComR dimer. Two symmetry related TPR domains of the apo ComR crystal packing form a low affinity dimer similar to the dimeric ComR/XIP/DNA complex. Residues K246 and E282 involved in the conserved salt bridge are highlighted in sticks.
(TIF)

S3 Fig SEC-MALLS analysis of the apo and peptide bound forms of ComR. The elution profiles of ComR alone at 8 mg ml-1 (A) or at 2 mg ml-1 in the presence of 1 mM XIP (B) are represented according to retention volume (in ml) with the refractive index (in mV) indicated on the left axis and the logarithm of molecular weight (Mw) on the right axis.
(TIF)

S1 Table Bacterial strains used in this study. (DOCX)

S2 Table Primers used in this study. (DOCX)

S3 Table Plasmids used in this study. (DOCX)

S4 Table X-ray diffraction data processing and refinement statistics. (DOCX)
