# Correction: Structural Insights into Streptococcal Competence Regulation by the Cell-to-Cell Communication System ComRS

**DOI:** 10.1371/journal.ppat.1006208

**Published:** 2017-02-14

**Authors:** Antoine Talagas, Laetitia Fontaine, Laura Ledesma-García, Johann Mignolet, Inès Li de la Sierra-Gallay, Noureddine Lazar, Magali Aumont-Nicaise, Michael J. Federle, Gerd Prehna, Pascal Hols, Sylvie Nessler

The third author's name is spelled incorrectly. The correct name is: Laura Ledesma-García. The correct citation is: Talagas A, Fontaine L, Ledesma-García L, Mignolet J, Li de la Sierra-Gallay I, Lazar N, et al. (2016) Structural Insights into Streptococcal Competence Regulation by the Cell-to-Cell Communication System ComRS. PLoS Pathog 12(12): e1005980. doi:10.1371/journal.ppat.1005980.
