# Supplementary figures and images for: Structural Insights into Streptococcal Competence Regulation by the Cell-to-Cell Communication System ComRS
Source: PLoS Pathog. 2016 Dec 1;12(12):e1005980. doi: 10.1371/journal.ppat.1005980 (PMC5131891; doi:10.1371/journal.ppat.1005980)

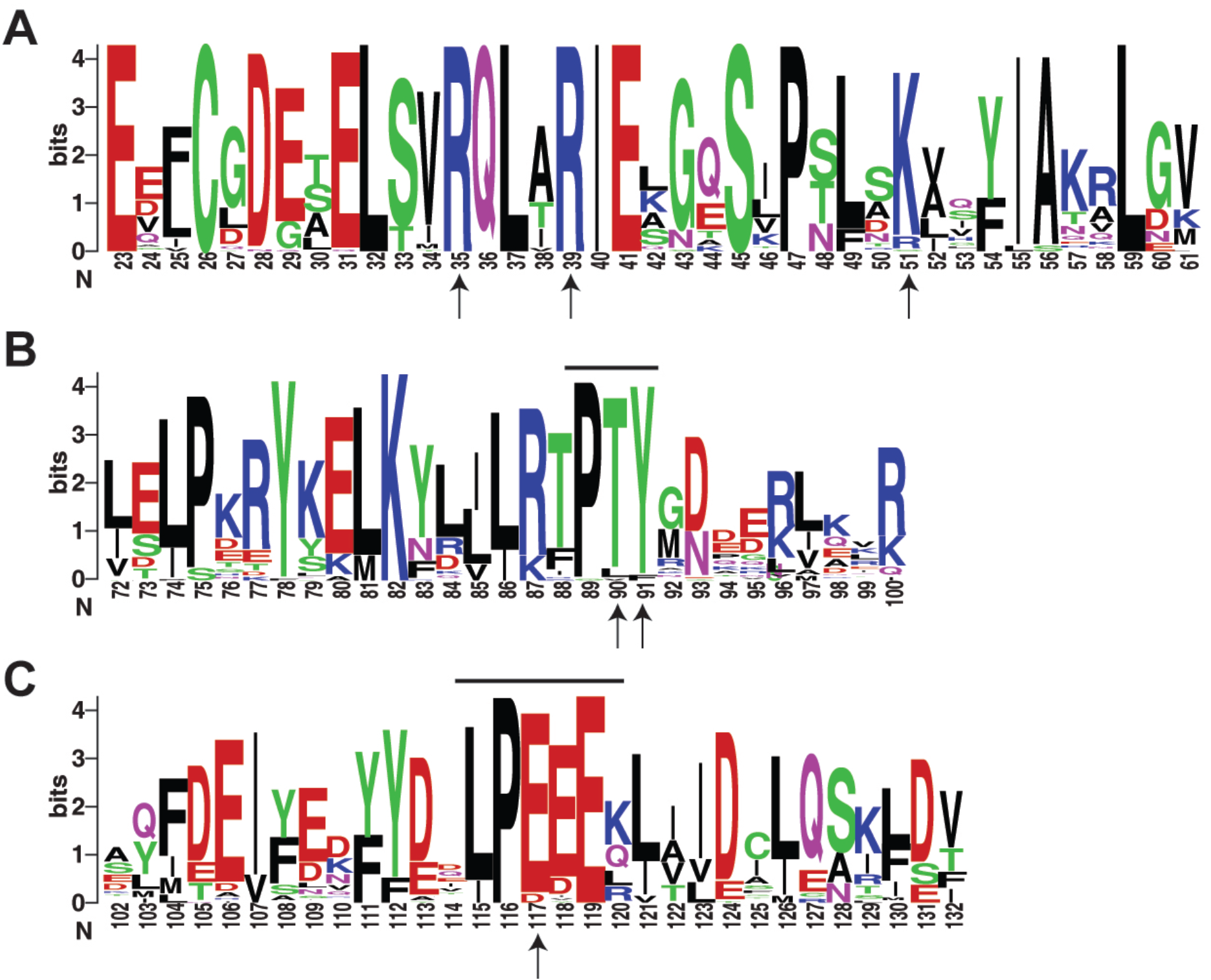

Supplement: S1 Fig — The HTH DNA binding domain (A), the PTY motif (B), and the LPEEE motif (C) are shown. Non-redundant representatives of ComR orthologues from each Streptococcus species (56 sequences used to generate a phylogenetic tree of ComR orthologues [35]) were aligned using BLAST on NCBI website and sequence logos were created using Weblogo (http://weblogo.berkeley.edu/logo.cgi) [68]. Bits represent the relative frequency of amino acids. X-axes refer to the position of residues in the ComR sequence of Streptococcus thermophilus LMD-9. Arrows highlight key residues. (TIFF) [file ppat.1005980.s001.tiff]

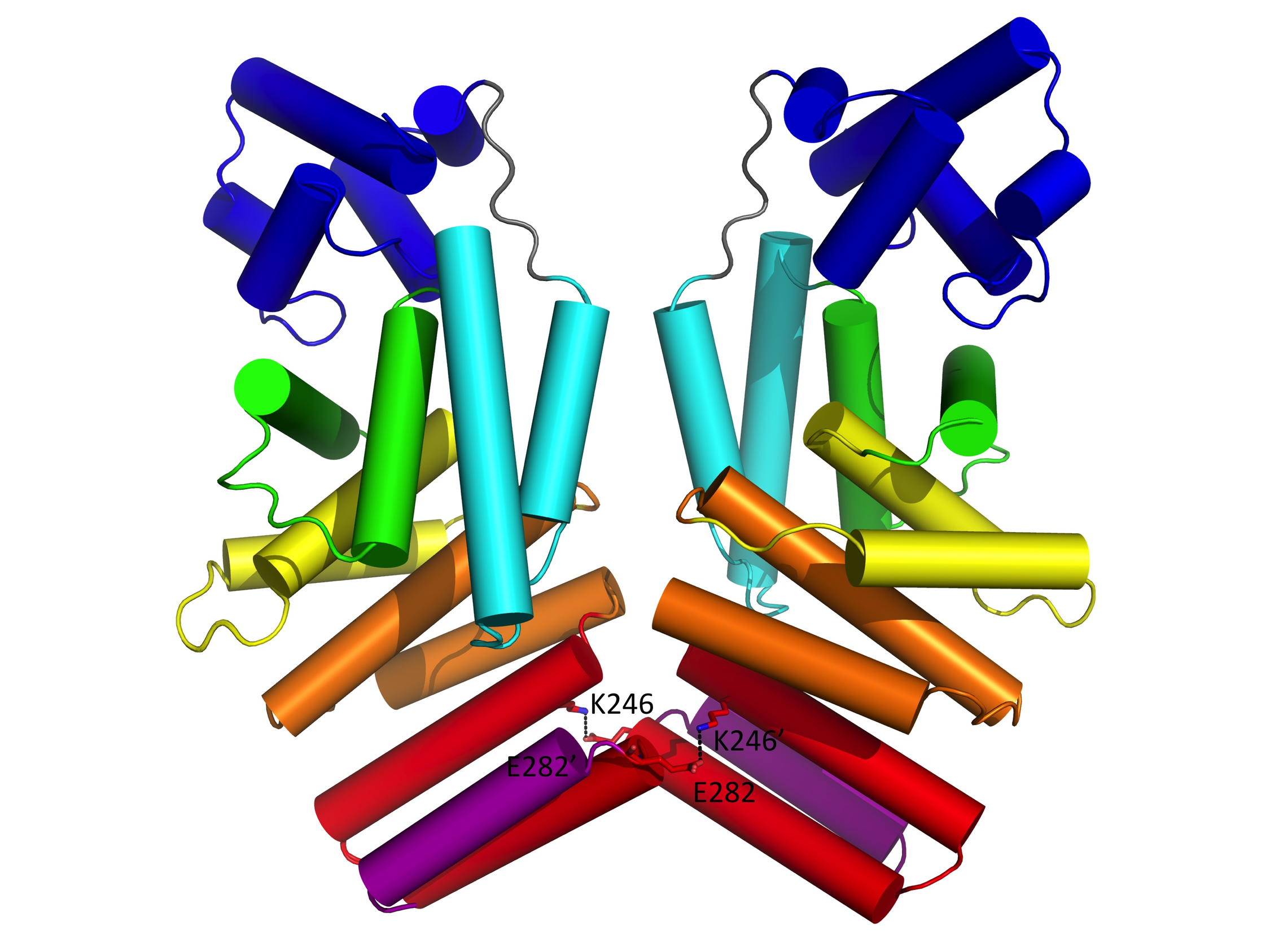

Supplement: S2 Fig — Two symmetry related TPR domains of the apo ComR crystal packing form a low affinity dimer similar to the dimeric ComR/XIP/DNA complex. Residues K246 and E282 involved in the conserved salt bridge are highlighted in sticks. (TIF) [file ppat.1005980.s002.tif]

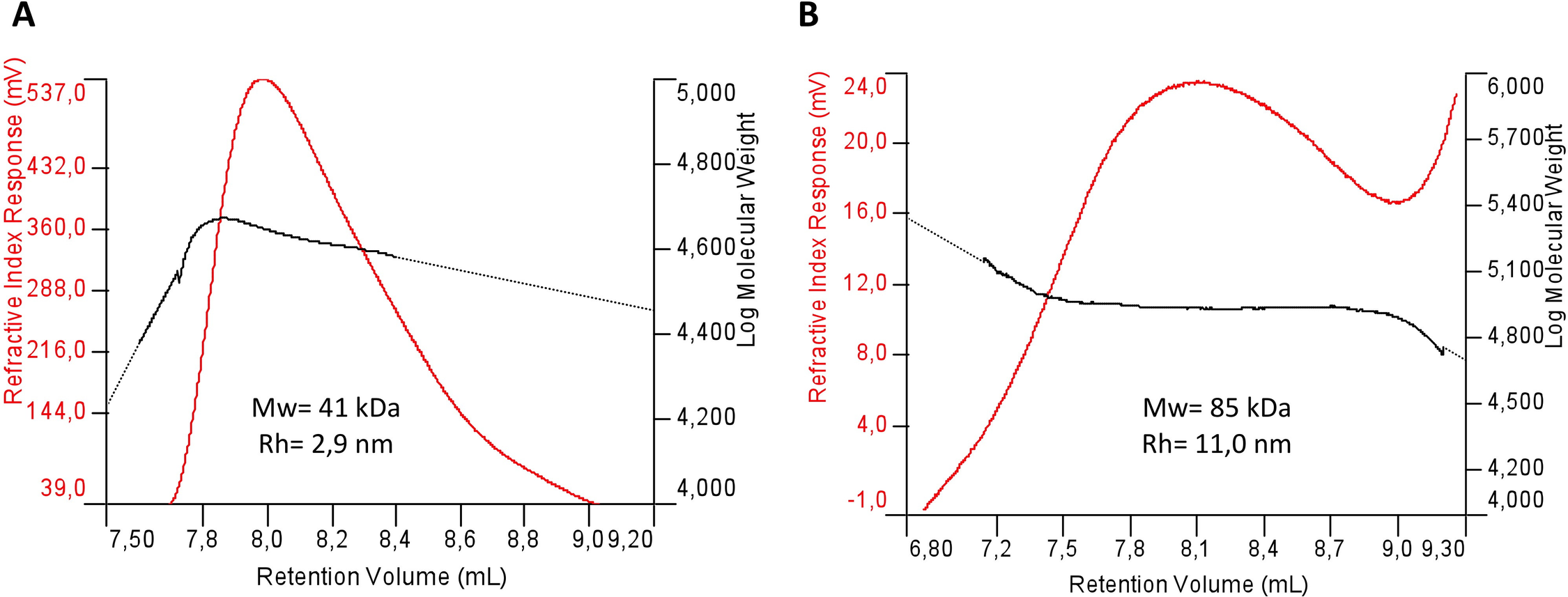

Supplement: S3 Fig — The elution profiles of ComR alone at 8 mg ml-1 (A) or at 2 mg ml-1 in the presence of 1 mM XIP (B) are represented according to retention volume (in ml) with the refractive index (in mV) indicated on the left axis and the logarithm of molecular weight (Mw) on the right axis. (TIF) [file ppat.1005980.s003.tif]
