# Supplementary material for: Structural Insights into Streptococcal Competence Regulation by the Cell-to-Cell Communication System ComRS
Source: PLoS Pathog. 2016 Dec 1;12(12):e1005980. doi: 10.1371/journal.ppat.1005980 (PMC5131891; doi:10.1371/journal.ppat.1005980)
Supplement: S1 Table — (DOCX) [file ppat.1005980.s004.docx]

**S1 Table.** Bacterial strains used in this study.

| **Strains** | **Genotype** | **Characteristic** | **Reference/source** |
| --- | --- | --- | --- |
| ***E. coli* strains** | |  |  |
| *E.coli* TOP10 | F^-^ *mcrA* Δ(*mrr-hsdRMS mcrBC*) φ80*lacZΔM15* Δ*lacΧ74 recA1 araD139* Δ(*ara-leu*) 7697 *galU galK* *rpsL* (Str^r^) *endA1 nupG λ-* |  | Invitrogen, CA |
| *E.coli* BL21-Gold | B F^-^ *ompT* *hsdS*(r_B_^-^m_B_^-^) *dcm^+^*Tet^r^ *gal endA* Hte |  | Stratagene, CA |
| ***S. thermophilus* strains** | | | |
| LF121 | LMD‐9 (*blpD‐blpX*)::P*_comS_‐luxA* |  | (1) |
| LF134 | LF121 Δ*comS*::P*_32_‐cat* | Cm^r^*^a^* | (1) |
| LF147 | LF121 *comR::comR*_E117A,E118A_, Δ*comS*::P*_32_‐cat* | Cm^r^*^a^* | This study |
| LF148 | LF121 *comR*::*comR*_E146A,D147A_, Δ*comS*::P*_32_‐cat* | Cm^r^*^a^* | This study |
| LF149 | LF121 *comR::comR*_T90A_, Δ*comS*::P*_32_‐cat* | Cm^r^*^a^* | This study |
| LF150 | LF121 *comR::comR*_Y91A_, Δ*comS*::P*_32_‐cat* | Cm^r^*^a^* | This study |
| LF151 | LF121 *comR::comR*_R92A_, Δ*comS*::P*_32_‐cat* | Cm^r^*^a^* | This study |
| LF152 | LF121 *comR::comR*_K100A_, Δ*comS*::P*_32_‐cat* | Cm^r^*^a^* | This study |
| LF153 | LF121 *comR::comR*_F171A,Y174A_, Δ*comS*::P*_32_‐cat* | Cm^r^*^a^* | This study |
| LF154 | LF121 *comR::comR*_K87A_, Δ*comS*::P*_32_‐cat* | Cm^r^*^a^* | This study |
| LF155 | LF121 *comR::comR*_K246A_, Δ*comS*::P*_32_‐cat* | Cm^r^*^a^* | This study |
| LL-09 | LF121 *comR::comR*_K87A,K246A_, Δ*comS*::P*_32_‐cat* | Cm^r^*^a^* | This study |

| *^a^*Cm^r^ indicates resistance to chloramphenicol. |
| --- |

1. Fontaine L, Goffin P, Dubout H, Delplace B, Baulard A, Lecat-Guillet N, et al. Mechanism of competence activation by the ComRS signalling system in streptococci. Mol Microbiol. 2013;87(6):1113-32.
