# Supplementary material for: Structural Insights into Streptococcal Competence Regulation by the Cell-to-Cell Communication System ComRS
Source: PLoS Pathog. 2016 Dec 1;12(12):e1005980. doi: 10.1371/journal.ppat.1005980 (PMC5131891; doi:10.1371/journal.ppat.1005980)
Supplement: S2 Table — (DOCX) [file ppat.1005980.s005.docx]

**S2 Table.** Primers used in this study

| **Oligonucleotide name** | | **Sequence and description** | | **Reference** |
| --- | --- | --- | --- | --- |
| **Primers used to introduce specific mutations in *comR-strep* in plasmid** pBADcomR_LMD‐9_-strep*^a^* | | | | |
| ComRT90A-Fw | 5’‐ GTTTCCA**GCT**TACAGAAACCCTGACAGAATAAAGTC ‐3’ | | Construction of pBADcomR_T90A_-strep | This study |
| ComRT90A-Rv | 5’‐ CAGGGTTTCTGTA**AGC**TGGAAACTTAATCAAACGATTC ‐3’ | |  | This study |
| ComRR92A-Fw | 5’‐ GTTTCCAACGTAC**GCT**AACCCTGACAGAATAAAGTC‐3’ | | Construction of pBADcomR_R92A_-strep | This study |
| ComRR92A-Rv | 5’‐ CAGGGTT**AGC**GTACGTTGGAAACTTAATCAAACGATTC‐3’ | |  | This study |
| Fw.ComRSth-K87A | 5’‐ gtttgatt**gct**tttccaacgtacagaaacc ‐3’ | | Construction of pBADcomR_K87A_-strep | This study |
| Rv.ComRSth-K87A | 5’‐ gttggaaa**agc**aatcaaacgattcttcatttc ‐3’ | |  | This study |
| Fw.ComRSth-K246A | 5’‐ gctac**gct**cctagtgtttttgtacttaag ‐3’ | | Construction of pBADcomR_K246A_-strep | This study |
| Rv.ComRSth-K246A | 5’‐ cactagg**agc**gtagctatataattgtgatttttc ‐3’ | |  | This study |
| **Primers used to create overlap PCR fragments containing the mutated *comR* alleles** | | | | |
| UpIntComRmut-Fw | | 5’‐ TTCTGTTTTAGGAACGATTTTGCTTACAGTTGC ‐3’ | Amplification of the upstream region to *comR* on strain LMD-9 | This study |
| UpIntComRmut-Rv | | 5’‐ ACGATTCTTCATTTCATAATATTCGTCAGGAATGG ‐3’ |  | This study |
| IntComRmut-Fw | | 5’‐ CTGACGAATATTATGAAATGAAGAATCG ‐3’ | Amplification of the mutated *comR* alleles on pBADcomR-strep plasmids | This study |
| IntComRmut-Rev | | 5’‐ TAAACCATCTGCCAATTTTCCTG ‐3’ |  | This study |
| DNIntComRmut_Fw | | 5’‐ CAGGAAAATTGGCAGATGGTTTATAGAAATG ‐3’ | Amplification of the downstream region of *comR* in strain LF134 | This study |
| DNIntComRmut_Rv | | 5’‐ AAATCATCAATAATAGCAGTATTGACCTGACTATTTGC ‐3’ |  | This study |
| ChSTER0316A | | 5’‐ TAAGAGTGCTATTGGTGTTCTCTTGC ‐3’ | Confirmation of transformants | (1) |
| ChSTER0316B | | 5’‐ TCATGGAATTTCACCTCAATTTCTTGC ‐3’ |  | (1) |
| **Primers used in EMSA experiments** | | | | |
| Cy3box1655directWT | | 5’‐ CAAAATCGAGTAGTGACATTTATGTCACTACTTTTTTGTT ‐3’ | Probe Cy3‐boxP*_ster_1655_*wt *S. thermophilus* LMD‐9 | (2) |
| box1655compWT | | 5’‐ AACAAAAAAGTAGTGACATAAATGTCACTACTCGATTTTG‐3’ |  | (2) |

*^a^*The mutant codon introduced in the primers is indicated in bold character.

**References :**

1. Fontaine L, Dandoy D, Boutry C, Delplace B, de Frahan MH, Fremaux C, et al. Development of a versatile procedure based on natural transformation for marker-free targeted genetic modification in Streptococcus thermophilus. Appl Environ Microbiol. 2010;76(23):7870-7.

2. Fontaine L, Goffin P, Dubout H, Delplace B, Baulard A, Lecat-Guillet N, et al. Mechanism of competence activation by the ComRS signalling system in streptococci. Mol Microbiol. 2013;87(6):1113-32.
