# Supplementary material for: Structural Insights into Streptococcal Competence Regulation by the Cell-to-Cell Communication System ComRS
Source: PLoS Pathog. 2016 Dec 1;12(12):e1005980. doi: 10.1371/journal.ppat.1005980 (PMC5131891; doi:10.1371/journal.ppat.1005980)
Supplement: S3 Table — (DOCX) [file ppat.1005980.s006.docx]

**S3 Table.** Plasmids used in this study

| **Plasmid name** | **Description** | **Characteristic** | **Reference** |
| --- | --- | --- | --- |
| pBADcomR_LMD‐9_-strep | pBADHisA derivative containing the translation fusion P*_araBAD_*-*comR*_LMD‐9_‐*strep*. | Ap^r^*^a^* | (1) |
| pBADcomR_E117A,E118A_- strep | pBADcomR_LMD‐9_-strep derivative containing the mutant *comR*_E117A,E118A_*-strep* allele under the control of P*_araBAD_*-. | Ap^r^*^a^* | This study |
| pBADcomR_E146A,D147A_-strep | pBADcomR_LMD‐9_-strep derivative containing the mutant *comR*_LMD‐9E146A,D147A_*-strep* allele under the control of P*_araBAD_*. | Ap^r^*^a^* | This study |
| pBADcomR_T90A_-strep | pBADcomR_LMD‐9_-strep derivative containing the mutant *comR*_T90A_*-strep* allele under the control of P*_araBAD_*. | Ap^r^*^a^* | This study |
| pBADcomR_Y91A_-strep | pBADcomR_LMD‐9_-strep derivative containing the mutant *comR*_Y91A_*-strep* allele under the control of P*_araBAD_*. | Ap^r^*^a^* | This study |
| pBADcomR_R92A_-strep | pBADcomR_LMD‐9_-strep derivative containing the mutant *comR*_R92A_*-strep* allele under the control of P*_araBAD_*. | Ap^r^*^a^* | This study |
| pBADcomR_K100A_-strep | pBADcomR_LMD‐9_-strep derivative containing the mutant *comR*_K100A_*-strep* allele under the control of P*_araBAD_*. | Ap^r^*^a^* | This study |
| pBADcomR_F171A,Y174A_-strep | pBADcomR_LMD‐9_-strep derivative containing the mutant *comR*_F171A,Y174A_*-strep* allele under the control of P*_araBAD_*. | Ap^r^*^a^* | This study |
| pBADcomR_K87A_-strep | pBADcomR_LMD‐9_-strep derivative containing the mutant *comR*_K87A_*-strep* allele under the control of P*_araBAD_*. | Ap^r^*^a^* | This study |
| pBADcomR_K246A_-strep | pBADcomR_LMD‐9_-strep derivative containing the mutant *comR*_K246A_*-strep* allele under the control of P*_araBAD_*. | Ap^r^*^a^* | This study |
| pBADcomR_K87A,K246A_-strep | pBADcomR_LMD‐9_-strep derivative containing the mutant *comR*_K87A,K246A_*-strep* allele under the control of P*_araBAD_*. | Ap^r^*^a^* | This study |

*^a^*Ap^r^ indicates resistance to ampicillin.

1. Fontaine L, Goffin P, Dubout H, Delplace B, Baulard A, Lecat-Guillet N, et al. Mechanism of competence activation by the ComRS signalling system in streptococci. Mol Microbiol. 2013;87(6):1113-32.
