# Supplementary material for: Structural Insights into Streptococcal Competence Regulation by the Cell-to-Cell Communication System ComRS
Source: PLoS Pathog. 2016 Dec 1;12(12):e1005980. doi: 10.1371/journal.ppat.1005980 (PMC5131891; doi:10.1371/journal.ppat.1005980)
Supplement: S4 Table — (DOCX) [file ppat.1005980.s007.docx]

**S4 Table: X-ray diffraction data processing and refinement statistics**

| Data processing statistics | Native data set  ComR/ComS/pComX | Native data set  ComR apo | SAD data set (SeMet)  ComR apo |
| --- | --- | --- | --- |
| Space group | C 1 2 1 | C 2 2 2 | C 2 2 2 |
| Unit-cell parameters (Å) | a=186.11 b=105.3; c=177.1 | a=61.82 b=141.68; c=78.55 | a=60.75; b=140.78; c=78.26 |
| Unit-cell angles (°) | α=90.0; β=98.3; γ=90.0 | α=90.0; β=90.0; γ=90.0 | α=90.0; β=90.0; γ=90.0 |
| Resolution range (Å)* | 50.0-2.57 (2.63-2.57) | 50.0-1.95 (2.06-1.95) | 50.0-2.78 (2.94-2.78) |
| No. of unique reflections | 27 134 (1940) | 25 537 (3943) | 16 117 (2384) |
| Completeness (%) | 99.0 (98.6) | 99.2 (96.1) | 98.3 (89.9) |
| Redundancy | 5.32 (3.0) | 10.9 (9.6) | 5.2 (5.0) |
| Mean I/σ(I) | 13.31 (1.81) | 12.53 (2.48) | 15.24 (5.50) |
| R_meas_(%)^a^ | 8.6 (58.0) | 11.9 (72.2) | 9.0 (28.9) |
| CC $\frac{1}{2}$^d^ | 99.8 (88.8) | 99.6 (83.3) | 99.7 (96.2) |
| Refinement statistics | Against native data set | Against native data set |  |
| Resolution range | 46.55-2.57 (2.66-2.57) | 45.95-1.95 (2.024-1.95) |  |
| No. of molecules/a.u. | 2 | 1 |  |
| R_work_(%)^b^ | 22.2 (33.56) | 18.0 (28.51) |  |
| R_free_(%)^c^ | 26.4 (35.62) | 21.5 (35.45) |  |
| Ramachandran |  |  |  |
| Favored (%) | 97.3 | 99.0 |  |
| Outliers (%) | 0.0 | 0.0 |  |
| R.M.S.D. |  |  |  |
| Bond lengths (Å) | 0.002 | 0.007 |  |
| Bond angles (°) | 0.420 | 0.753 |  |
| Chirality | 0.033 | 0.048 |  |
| Planarity | 0.002 | 0.004 |  |
| Dihedral | 15.878 | 17.2 |  |
| Average B, all atoms (Å^2^) | 73.22 | 38.0 |  |

* Numbers in parentheses represent values in the highest resolution shell.

**a** Rmeas = ∑hkl [N/N-1]^1/2^∑i |Ii(hkl) - <I(hkl)>| / ∑hkl∑i Ii(hkl) where N is the multiplicity of a given reflection, Ii(hkl) is the integrated intensity of a given reflection and <I(hkl)> is the mean intensity of multiple corresponding symmetry-related reflections.

**b** Rwork = ∑ ||Fobs| - |Fcalc|| / ∑ |Fobs|, where |Fobs| and |Fcalc| are the observed and calculated structure factor amplitudes respectively.

**c** Rfree is the same as Rwork but calculated with a 20% subset of all reflections that was never used in refinement.

**^d^** CC_1/2_ = percentage of correlation between intensities from random half‐dataset.
